# Psychometric properties of the Albanian version of chewing-function questionnaire CFQ-ALB

**DOI:** 10.1186/s12955-016-0437-3

**Published:** 2016-03-03

**Authors:** Venera Bimbashi, Gloria Staka, Asja Čelebić, Flurije Hoxha, Kujtim Shala, Nikola Petričević

**Affiliations:** Department of Prosthodontics, Dental School, Faculty of Medicine, University of Prishtina, Republic of Kosovo, Rrethi i Spitalit p.n., 10000 Prishtina, Republic of Kosovo; Department of Prosthodontics, Dental School, Faculty of Medicine, University of Prishtina and University Dentistry Clinical Center of Kosovo, Prishtina, Republic of Kosovo; Department of Prosthodontics, School of Dental Medicine, University of Zagreb and Clinical Hospital Centre, Zagreb, Croatia; Department of Prosthodontics, School of Dental Medicine, University of Zagreb, Zagreb, Croatia

**Keywords:** Chewing function questionnaire, Quality of life, Oral health, Psychometric properties

## Abstract

**Background:**

The new Chewing Function Questionnaire (CFQ) was lately developed in Croatia to measure the chewing ability in prosthodontic patients, as a one-dimensional instrument consisting of 10-items. Therefore, the objective of this study was to develop an Albanian version of the CFQ questionnaire and to test its psychometric properties in a new typical environment among the Kosovo population.

**Materials and methods:**

The original version of CFQ questionnaire was translated and cross-culturally adapted from the English language into Albanian in accordance with international guidelines. Its validity (construct, convergent and discriminative) and internal consistency (reliability) were tested in 205 participants. Test-retest reliability was evaluated in 61 subjects with natural teeth, and responsiveness was evaluated in 51 prosthodontic patients with treatment needs.

**Results:**

Internal consistency of CFQ-ALB indicated excellent agreement, with Cronbach’s alpha values of 0.974 and average inter-item correlation of 0.792. Intraclass correlation coeficinets for test-retest were found without significant differences by 95 % of confidence intervals (*p* > 0.05). Construct validity was supported by a single factor that accounted for 81.711 % of the variance observed. Convergent validity was supported by the association between self-reported general satisfactions with chewing and CFQ summary scores. Discriminat validity was supported as statistically significant differences were observed between pre-defined groups. Responsiveness was confirmed by the significant difference between baseline summary scores and the after treatment scores; the mean change was 15.57 (SD =2.49) (*p* < 0.001).

**Conclusion:**

The obtained results suggest excellent psychometric properties of the CFQ-ALB questionnaire for determining chewing function in the Republic of Kosovo.

## Background

Chewing is one of the essential functions of the stomatognathic system. Teeth are necessary for food diminution. Some studies investigated how many teeth in oral cavity are necessary for a good chewing ability and the association of tooth loss with general and oral health [1].

The purpose of a therapy of fully or partially edentulous patients with removable or fixed dentures is to restore their chewing function, speaking ability, and to improve their facial appearance and overall quality of life. Adequate chewing function should enhance both, general health and quality of life [2–5]. Chewing difficulties are more commonly found in elderly population due to physiological ageing, which may be related to teeth wear and/or to a loss of periodontal tissues, increased mobility of residual teeth and/or teeth loss as well as decrease of residual alveolar bone [6]. Even individuals with natural teeth may have problems with chewing of certain food due to restorations, tooth wear, oral mucosa diseases or temporomandibular disorders [7–14].

A number of objective methods have been attempted to evaluate chewing performance, all of which require trained staff with specific materials and instruments. Additionally, the aforementioned methods are considered as expensive procedures and impractical for everyday practice due to considerable consumption of time. Nevertheless, the measurement of the chewing function has been widely studied, by classical single sieving test and multiple sieve methods with natural test food (salted peanuts, carrots, almond, soya bean, coffee beans, and so forth) or artificial test food (gelatin, silicone impression materials, mixture of calcium carbonate, irreversible hydrocolloid/alginate). Also, a colorimetric determination has been used which evaluated chewing performance by using color changeable chewing gums [15–17].

In literature one of the most commonly accepted instruments for measuring the impact of oral problems on oral health related to quality of life (OHRQoL) is the cross-culturally adapted questionnaire with excellent psychometric properties: Oral Health Impact Profile (OHIP) (3). The Mandibular Function Impairment Questionnaire (MFIQ), Jaw Functional Limitation Scale (JFLS) and General Oral Health Assessment Index (GOHAI) are multidimensional questionnaires which contain items related to dental tissues and their impact on function of the lower jaw and its difficulty on chewing different types of food, which also contain psychosocial components [18–20].

Previous studies utilized different questionnaires for assessing chewing function, where individuals were asked to assess using a visual-analog scale, their chewing ability of different kind of food. One of those questionnaires was the Food Intake Questionnaire, which assesses the chewing ability categorizing different foods, mostly with Japanese food and their level of chewiness [21]. However, there are no data for the measurement of its psychometric properties in the original study, or in other populations. Furthermore, Sato et al., had used a questionnaire for self-evaluation of chewing function of complete denture wearers in the Japanese population, which is related to chewing different types of Japanese food [22]. Kazuyoshi et al., in 2009 have tested the psychometric properties of the same questionnaire for partial removable denture wearers [23]. Nevertheless, the psychometric properties of the same questionnaire were not further tested in other cultural environments because Japanese food is not an everyday food and widely used.

The Jaw Functional Limitation Scale (JFLS) questionnaire was developed in 2008 by the Swedish researchers Ohrbach et al., and was used in patients with temporomandibular disorders [19]. There are two versions of the above questionnaire; the long version containing 20 questions and a short version with 8 questions. Both questionnaires measure constructs limitations when chewing, lower jaw functional mobility, verbal and emotional expression.

Recently the new Chewing Function Questionnaire (CFQ), designed to measure the chewing ability in prosthodontic patients, was developed in Croatia, as a simple and one-dimensional instrument consisting of 10-items [24]. The original instrument of the CFQ consists of items related to textures of different types of food, such as softness, stickiness, hardness, and so forth. Also to difficulties when biting different foods (food incision) or feeling insecure while chewing, and food catching or remaining stuck in teeth or dentures during or after meals. However, the CFQ is appropriate for western food cultural milieu, and not for the vegetarians or individuals with different eating habits [24].

It is important for both patients and dentists to make a quality assessment of all types of denture without using any special equipment or without consuming a lot of time. The most important part of measuring chewing function efficiency should be based on the patients’ perceptions. The Republic of Kosovo is comprised mainly of ethnic Albanian population, with Albanian being the country’s official language. Since this measure has not been validated in the Albanian language, the aim of this study was to develop the Albanian language version of the Chewing Function Questionnaire and to test its psychometric properties in a typical cultural environment in the Republic of Kosovo.

## Methods

### Participants

A sample of 205 subjects, aged from 19 to 86 years participated in this study. The study was approved by the Ethics Committee of the University Dentistry Clinical Center of Kosovo. Informed consent was obtained from each subject participating in this study. The sample was divided into four groups. Group A comprised of randomly selected employees of Raiffeisen Bank in Prishtina, with natural teeth (NT, *n* = 36). Group B comprised of dental students with natural teeth (NT, *n* = 61), who were examined by a specialist of prosthodontics; the examination results indicated that none of the dental students required dental treatment. Group C a convenience sample comprised prosthodontics patients (*n* = 57) who already had prosthetic restorations that were not older than one year, and they were satisfied with them. Twenty-seven of them had fixed partial dentures (FPD) (C1; *n* = 27) and thirty had removable dentures (RD) (C2; *n* = 30). Group D included prosthodontics patients (D; *n* = 51) asking for prosthodontic treatment. Specialist of prosthodontics assessed that 31 patients (D1) needed FPD and 20 patients needed RD (D2). Groups C and D were selected at the Department of Prosthodontics, Dental School, Faculty of Medicine, University of Prishtina and Private Dental Clinic GS, Prishtina in Kosovo. Both prosthodontic sample groups (C,D) were interviewed, whereas the two first sample groups (A,B) with natural teeth self-administered the CFQ questionnaire and were supervised by three dentists. For each question, subjects were asked how frequently they have experienced chewing difficulties, using ordered coded pseudo continuous response categories; 0 = never, 1 = hardly ever, 2 = occasionally, 3 = fairly, 4 = very often or extreme difficulties. Zero indicates the absence of any chewing difficulties and higher scores indicate more impaired chewing function. Ten items of CFQ can sum up to give a CFQ summary score, with lower scores representing satisfaction with their chewing function and higher scores representing impaired chewing function. Besides the CFQ questionnaire, the subjects also answered another question related to their general satisfactions with chewing, using a 5-point Likert scale that ranged from 5 (excellent) to 1 (unsatisfactory).

### Translation and cultural adaption

The original version of the CFQ was translated from the English language into Albanian according to the accepted techniques, already used in earlier validation studies of Oral Health Related to Quality of Life [5, 25, 26]. The original edition which contained 10 items was translated into the Albanian language by a qualified and experienced translator who possesses excellent knowledge of dental terminology and interpretation. Furthermore, two other dentists with excellent proficiency in English were also involved for the translation of several terms. After being translated, the CFQ-ALB was edited by three other dentists, (Dental School, Faculty of Medicine, University of Prishtina), with an excellent knowledge of both Albanian and English language. The translation was done individually and then the last version was incorporated into the final version. Further, the final version was back-translated into English by another qualified freelance translator, in collaboration with two other Kosovar dentists with excellent knowledge of English. The final version translated back to English was evaluated independently by a professor from the Dental School, University of Zagreb, with an excellent proficiency of English and also by a native English language speaker. There were no significant differences between the back translated and the original questionnaire. Considering that there were no dissimilarities in the implication of the items which were observed, the final translation was considered to be adequate for its further use. To test the clarity of the CFQ items in the Albanian language, a pilot study was performed with 20 prosthodontic patients (age 25 to 59 years). Patients were asked whether they had any difficulties understanding and answering the items of the questionnaire. All items were understood clearly, without any difficulties.

### Data analysis

#### Reliability

The internal consistency of the questionnaire and the test-retest reliability were assessed. Internal consistency of the CFQ was assessed by the Cronbach’s alpha coefficient and inter-item correlation for all groups and also independently for each group [27]. All 205 subjects were included while testing the internal consistency. The Cronbach’s alpha values > 0.75 indicates excellent outcome; whereas, values between 0.40 and 0.75 are considered to have fair to good reliability, and values of < 0.40 are considered as poor reliability [27]. The test-retest reliability was measured only in group B (Table 1). Participants completed the questionnaire two times within a two-week period; meanwhile no orofacial or dental treatments were conducted. The Intraclass correlation coefficient – ICC according to the Fleiss’s and Shrout were computed [28]. The high values of ICC > 0.80 indicated excellent agreement, the range from 0.61 to 0.80 indicated good agreement, 0.41- 0.60 moderate agreement and the values <0.40 indicated poor agreement [29].

**Table 1:** Sample overview (number, age, and gender), data-collection methods, sampling strategies and research purpose – CFQ in Albanian language

Sample	Sample type	Data collection	*N*	Age mean (SD)	Age range	% women	Type of investigation
(A) General population Natural teeth - NT (*n* = 36)	Random	Questionnaire self-administered supervised	36	33.58 (6.11)	25–44	50.00	Internal consistency Convergent validity Discriminate validity
(B) Dental Students Natural teeth – NT (*n* = 61)	Consecutive	Questionnaire self-administered supervised	61	22.13 (0.46)	21–23	60.65	Internal consistency Test-retest reliability Convergent validity Discriminate validity
(C) Prosthodontic patients (*n* = 57) (C1, C2) (C1) Fixed Partial Dentures (FPD) (*n* = 27) (C2) Removable dentures (RDs) (*n* = 30)	Convenience	Questionnaire interview	57	49.92 (14.52)	20–86	49.12	Internal consistency Convergent validity Discriminate validity
(D) Prosthodontic patients with a treatment need (*n* = 51) (D1, D2) (D1) Fixed Partial Dentures (FPD) (*n* = 31) (D2) Removable dentures (RDs) (*n* = 20)	Convenience	Questionnaire interview	51	49.50 (16.13)	19–73	50.98	Internal consistency Convergent validity Discriminate validity Responsiveness

A General population- Natural teeth; Raiffaisen Bank – Prishtina

B Dental Students - Natural teeth; Dental School, Faculty of Medicine, University of Prishtina

C,D Prosthodontic patients - Department of Prosthodontics, Dental School, Faculty of Medicine University of Prishtina and Private Dental Clinic GS, Prishtina, Kosovo

### Validity

Validity was investigated by evaluating construct, convergent and discriminat validity [30]. All subjects were included. Exploratory factor analysis (EFA) was used to assess the factor structure of the CFQ-ALB. The factor extraction criterion was eigen value more than 1. An item is considered to have loaded onto a factor when the factor loading exceeded 0.30. Due to the small number of participants, we did not perform a confirmatory factor analysis. The Bartlett’s test of sphericity, scree plot, and the Kaiser-Mayer-Olkin (KMO) test were used [31, 32]. Convergent validity was determined by Spearman’s rank correlation between self-reported general satisfaction with chewing’ and the CFQ summary scores. Discriminant validity was evaluated using one-way analysis of variance and Sheffe post-hoc tests, by determining whether patients with natural teeth (A, B), fixed partial dentures (C1, D1) and removable dentures (C2, D2) significantly different from one another. The natural teeth group (A,B) was expected to have lower CFQ summary scores in comparison to fixed partial denture group (C1, D1) and the removable denture group (C2, D2).

### Responsiveness

Responsiveness was tested on fifty-one prosthodontic patients with a treatment need [33]. They completed the questionnaire twice; prior to the treatment and a month after they had received new dentures. One month period was considered acceptable for the patients to adapt to their new prosthodontic restorations. Responsiveness was tested using the paired *t*-test and by calculating the standardized response mean and the effect size [34]. According to Cohen, the effect size >0.80 is considered large, 0.50 moderate and the effect size < 0.20 is considered small. Satisfactory changes, i.e. improvement of the chewing ability after receiving new prosthodontic restoration was expected. The standardized effect size was computed as following: (baseline score of CFQ - after treatment score)/standard deviation of the baseline CFQ score [35].

The survey data were organized and analyzed using SPSS 19 for Windows (SPSS Inc., Chicago, Illinois, USA) and MS Excel (Microsoft Office, Windows 2007, USA). The significance level was set at 95 % probability (*p* < 0.05).

## Results

The overview of the present study, data-collection methods with sampling strategies and research purpose – CFQ in the Albanian language in the Republic of Kosovo are accessible in Table 1.

### Reliability

The corrected item-total correlation, part of the internal consistency of the CFQ in the Albanian language ranged from 0.646 to 0.924 (Table 2). The highest coefficients were obtained for two items “Have you had any difficulty chewing apples/raw carrots or foods of similar consistency?” and “Have you had any difficulties chewing biscuits, crackers, tea biscuits or foods of similar consistency?” and the lowest coefficient was obtained for the item “Have you noticed food catching or food remaining stuck in your teeth or dentures during or after meals?”. If items were deleted one by one, the Cronbach’s alpha would not increase and it ranged between 0.968 and 0.971 (Table 2). The highest correlation was found between “Have you had any difficulties chewing biscuits, crackers, tea biscuits or foods of similar consistency?” and “Have you had any difficulty chewing fresh bread, doughnuts or foods of similar consistency?” The weakest correlation was found between items “Have you noticed food catching or food remaining stuck in your teeth or dentures during or after meals?” and “Have you had any difficulty chewing bacon/smoked ham/baked or fried firm meat or foods of similar consistency?” (Table 3). The obtained results suggest good internal consistency for the CFQ-ALB. The Cronbach’s alpha coefficients for the overall sample, as well as for each group are shown in Table 4. The Cronbach’s alpha coefficient calculated for all subjects was 0.974 and the Inter-item correlation - IIC was 0.792. For the prosthodontic patients in need of treatment (D1, D2), Cronbach’s alpha coefficient was 0.946 and the IIC was 0.634. For the prosthodontic patients (C1, C2) Cronbach’s alpha coefficient was 0.968 and the IIC was 0.761, and for the natural teeth (Sample A, B) the Cronbach’s alpha coefficient was 0.912 and the IIC 0.534. These results confirmed excellent internal consistency. Results of intraclass correlation coefficients are presented in Table 5. Test-retest reliability was supported.

**Table 2:** Results obtained for the CFQ – ALB

CFQ item	Mean	SD	Corrected item-total correlation	Cronbach's Alpha if item deleted	Factor loading
1. Have you had any difficulty chewing apples/raw carrots or foods of similar consistency?	1.22	1.17	0.924	0.968	0.941
2. Have you had any difficulty chewing bacon/smoked ham/baked or fried firm meat or foods of similar consistency?	1.73	1.39	0.887	0.970	0.908
3. Have you had any difficulties chewing biscuits, crackers, tea biscuits or foods of similar consistency?	1.11	1.06	0.924	0.969	0.942
4. Have you had any difficulty chewing fresh bread, doughnuts or foods of similar consistency?	1.01	1.03	0.917	0.969	0.936
5. Have you had any difficulty chewing nuts/walnuts/almonds/macadamia or similar food?	1.34	1.24	0.912	0.969	0.931
6. Have you had any difficulty chewing lettuce, raw cabbage or similar food?	1.03	1.06	0.909	0.969	0.930
7. Have you felt insecure when you are chewing?	1.22	1.15	0.901	0.969	0.923
8. Have you had any difficulty when biting different foods (food incision)?	1.27	1.20	0.886	0.970	0.910
9. Have you noticed food catching or food remaining sticked in your teeth or dentures during or after meals?	1.80	1.00	0.646	0.977	0.694
10. Have you had any difficulty chewing chewing-gum?	1.42	1.42	0.874	0.971	0.896

**Table 3:** Inter-Item Correlation statistics of the CFQ-ALB

	Q1	Q2	Q3	Q4	Q5	Q6	Q7	Q8	Q9	Q10
Q1	1.000									
Q2	0.835	1.000								
Q3	0.886	0.840	1.000							
Q4	0.865	0.823	0.922	1.000						
Q5	0.866	0.849	0.879	0.873	1.000					
Q6	0.860	0.785	0.867	0.882	0.853	1.000				
Q7	0.868	0.802	0.840	0.850	0.839	0.865	1.000			
Q8	0.847	0.798	0.842	0.809	0.812	0.843	0.854	1.000		
Q9	0.631	0.567	0.584	0.593	0.585	0.606	0.623	0.600	1.000	
Q10	0.814	0.869	0.812	0.803	0.814	0.807	0.774	0.790	0.597	1.000

**Table 4:** Internal consistency (Cronbach’s alpha values) for all samples (A, B, C and D) of CFQ-ALB scores

	*N*	Cronbach α	Mean IIC
Natural teeth Group A, B	97	0.912	0.534
Prosthodontic patients Group C	57	0.968	0.761
Prosthodontic patients with treatment need Group D	51	0.946	0.634
All subjects	205	0.974	0.792

*IIC* Inter Item Correlation

**Table 5:** Test-retest reliability for each item and summary score CFQ-ALB, tested on 61 subjects, with natural teeth (sample B)

Question	ICC	Mean difference	95 % confidence interval of the difference	t	*P*
Q1	0.43	−0.0327	−0.14 ± 0.07	−0.63	0.532 NS
Q2	0.79	0.0327	−0.07 ± 0.14	0.63	0.532 NS
Q3	0.66	0.0000	−0.08 ± 0.08	0.00	1.000 NS
Q4	0.78	0.0000	−0.04 ± 0.04	0.00	1.000 NS
Q5	0.76	0.0327	−0.04 ± 0.11	0.81	0.419 NS
Q6	0.76	0.0163	−0.01 ± 0.04	1.00	0.321 NS
Q7	0.76	0.0327	−0.06 ± 0.12	0.70	0.484 NS
Q8	0.80	0.0327	−0.04 ± 0.11	0.81	0.419 NS
Q9	0.51	0.0491	−0.16 ± 0.26	0.47	0.643 NS
Q10	0.81	−0.0163	−0.04 ± 0.01	1.00	0.321 NS
Summary score	0.90	0.1475	−0.11 ± 0.40	1.14	0.260 NS

*NS* not significant (*p* > 0.05), *ICC* Interclass Correlation Coefficients

### Validity

**Fig. 1:**
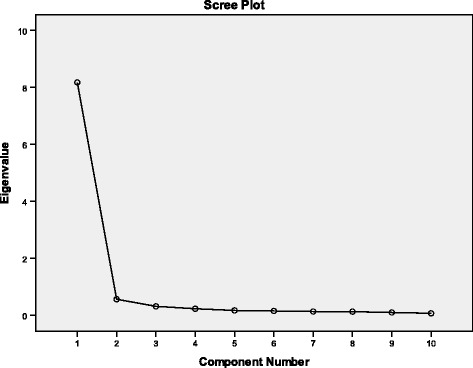
Scree Plot – CFQ

The results of the factor analysis for CFQ is given in Table 2. Exploratory factor analysis revealed one-factor structure on the basis of eingenvalue >1 and accounted for the 81.711 % of the variance (Scree plot; Fig. 1). The one-dimensional model results are considered acceptable corresponding to other published studies [24, 36]. Convergent validity was established by the strong relationship between the self-reported general satisfaction with chewing score and the CFQ-ALB summary score, as well as by statistically significant relationship between general satisfaction with chewing function and each item of the instrument (Table 6). The results of discriminant validity are presented in Table 7. Statistically significant differences between all sample groups was observed for each question, as well as for the CFQ summary score (*p* < 0.01). The lowest scores pertaining to each individual question and the CFQ summary score in the NT samples (A,B), followed by the FPD (C1,D1) with the highest scores in the RD (C2,D2).

**Table 6:** Convergent validity by Spearman’s rank correlation between self reported general satisfaction with chewing and the CFQ summary score for all samples (A, B, C and D)

Question	General satisfaction with chewing ability
Q1	0.842^**^
Q2	0.814^**^
Q3	0.857^**^
Q4	0.852^**^
Q5	0.821^**^
Q6	0.797^**^
Q7	0.824^**^
Q8	0.828^**^
Q9	0.614^**^
Q10	0.800^**^
Summary score	0.884^**^

^**^Correlation is significant at the 0.01 level (2-tailed)

**Table 7:** Discriminat validity by comparison of each question and the CFQ summary scores between all sample groups: with natural teeth (sample A,B), fixed partial dentures (C1,D1) and removable dentures wearers (C2, D2)

Question	Group	X	SD	F	*P*
Q1	Natural teeth	0.37	0.62	102.39	<0.001
	Fixed partial denture	1.75	0.91		
	Removable denture	2.25	1.06		
Q2	Natural teeth	0.68	0.76	160.18	<0.001
	Fixed partial denture	2.09	1.06		
	Removable denture	3.31	0.84		
Q3	Natural teeth	0.35	0.60	101.53	<0.001
	Fixed partial denture	1.54	0.83		
	Removable denture	2.08	0.91		
Q4	Natural teeth	0.25	0.50	110.44	<0.001
	Fixed partial denture	1.46	0.85		
	Removable denture	1.96	0.89		
Q5	Natural teeth	0.51	0.74	88.46	<0.001
	Fixed partial denture	1.68	0.95		
	Removable denture	2.53	1.14		
Q6	Natural teeth	0.26	0.60	99.98	<0.001
	Fixed partial denture	1.58	0.84		
	Removable denture	1.90	0.90		
Q7	Natural teeth	0.48	0.71	64.71	<0.001
	Fixed partial denture	1.65	0.99		
	Removable denture	2.14	1.11		
Q8	Natural teeth	0.46	0.75	77.26	<0.001
	Fixed partial denture	1.77	1.09		
	Removable denture	2.25	0.96		
Q9	Natural teeth	1.37	0.82	24.29	<0.001
	Fixed partial denture	2.00	1.04		
	Removable denture	2.41	0.88		
Q10	Natural teeth	0.30	0.62	267.57	<0.001
	Fixed partial denture	1.68	0.87		
	Removable denture	3.25	0.82		
Summary score	Natural teeth	5.03	5.06	144.16	<0.001
	Fixed partial denture	17.21	8.32		
	Removable denture	24.10	7.85		

One-Way Anova, Sheffe post hoc

### Responsiveness

The responsiveness was confirmed by the significant difference between the baseline summary scores and the after treatment scores (Table 8). The mean change between the baseline and the after treatment summary scores was 15.57 (SD = 2.49) (*p* < 0.001, Table 8). The effect size for the CFQ-ALB summary score was 2.03.

**Table 8:** Responsiveness of the CFQ-ALB tested on the 51 prosthodontic patients (sample D1 and D2)

	Before treatment	After treatment		
Item	x ± SD	X ± SD	T	*p*
Q1	2.67 ± 0.93	1.06 ± 0.54	16,52	0.001*
Q2	3.06 ± 1.01	1.55 ± 0.94	13,75	0.001*
Q3	2.35 ± 0.84	0.92 ± 0.52	12,68	0.001*
Q4	2.27 ± 0.80	0.84 ± 0.50	11,96	0.003*
Q5	2.76 ± 1.03	1.25 ± 0.82	11,94	0.001*
Q6	2.31 ± 0.84	0.94 ± 0.54	10,70	0.001*
Q7	2.65 ± 0.93	0.90 ± 0.64	13,33	0.001*
Q8	2.69 ± 1.05	1.00 ± 0.66	11,70	0.001*
Q9	2.80 ± 0.75	1.04 ± 0.63	14,62	0.001*
Q10	2.76 ± 1.01	1.25 ± 1.02	12,25	0.001*
Summary score	26.33 ± 7.67	10.76 ± 5.18	17,09	0.000*

*p < 0.001; (df) =50

## Discussion

To our understanding, the present study represents the first appraisal of the psychometric properties of the CFQ in the Albanian language. It is very important for dentists to evaluate the patient reported outcomes of prosthodontic therapy. There are some instruments which are widely used for the assessment of the OHRQoL, such as the short form OHIP-14 questionnaire and the original OHIP-49 [3, 5, 13, 37]. However, the OHIP questionnaire does not give a dentist full insight about the chewing function status or a change elicited by a provided treatment. To have an appropriate instrument for chewing ability assessment in the Republic of Kosovo it was decided to translate into Albanian language according to the appropriate principles and to test the translated CFQ’s psychometric properties in the new cultural environment.

In this study the psychometric properties of the Albanian language version of the CFQ questionnaire were assessed, which was recently developed [24] based on patients’ outcomes and with regards considering chewing function. The instrument was translated from English version into Albanian language consistent with international practices and it was adapted into the new cultural environment [25]. The psychometric properties of the CFQ-ALB were satisfactory.

The use of internationally validated instruments is strongly suggested to allow international assessment of the results of national studies. Hence, the strength of study includes all necessary steps and pilot testing, as suggested for an appropriate validation of the instruments. Consequently, good psychometric properties of the CFQ-ALB will allow further research regarding chewing normative values in general populations and/or prosthodontics patient groups of the Republic of Kosovo. As diversity in norms and values across cultures may exist, the trans-cultural comparisons of CFQ-ALB will also allow correlation of the obtained results from Kosovo with the results obtained from more developed countries with different cultures. Few limitations of the study must be considered. First, expert group ratings were not performed in this study. Second, the test-retest reliability was performed with a consecutive rather than random sample, which was compromised by including dental students with natural teeth and not including participants with lower education. Third, generalizability of the findings may be limited as group A comprised bank employees who are considered to be highly educated and who generate higher income. Therefore, a potential variety bias in our sample cannot be completely excluded.

## Conclusion

The original version of CFQ was translated and adapted effectively into a new cultural environment in the Albanian language, in the Republic of Kosovo. The obtained results confirmed the reliability of the instrument for determining chewing function in research and clinical trials in the Republic of Kosovo. The CFQ-ALB could set aside the use of the questionnaire for controlling the quality appraisal of the prosthodontic therapy, improving chewing ability achieved after treatment, instantly at clinic without the use of any special equipment or time consumption. It is short and practical, simple to use and it is appropriate for the measurement of a self-perceived chewing function, which could also be used for assessing the OHRQoL in longitudinal and cross-sectional studies.

## Abbreviations

CFQ: Chewing Function Questionnaire
CFQ-ALB: Chewing Function Questionnaire in Albanian
FPD: Fixed Partial Denture
ICC: Intraclass Correlation Coefficients
IIC: Inter-Item Correlation.
NT: Natural Teeth
OHIP: Oral Health Impact Profile
OHRQoL: Oral Health Related to Quality of Life
RD: Removable Denture
